# Local and Circulating Endothelial Cells Undergo Endothelial to Mesenchymal Transition (EndMT) in Response to Musculoskeletal Injury

**DOI:** 10.1038/srep32514

**Published:** 2016-09-12

**Authors:** Shailesh Agarwal, Shawn Loder, David Cholok, Joshua Peterson, John Li, David Fireman, Christopher Breuler, Hsiao Sung Hsieh, Kavitha Ranganathan, Charles Hwang, James Drake, Shuli Li, Charles K. Chan, Michael T. Longaker, Benjamin Levi

**Affiliations:** 1 Burn/Wound and Regenerative Medicine Laboratory, Department of Surgery, University of Michigan, Ann Arbor, MI 48109, USA; 2 Department of Surgery, Stanford University, Stanford, CA 94305, USA.

## Abstract

Endothelial-to-mesenchymal transition (EndMT) has been implicated in a variety of aberrant wound healing conditions. However, unambiguous evidence of EndMT has been elusive due to limitations of *in vitro* experimental designs and animal models. *In vitro* experiments cannot account for the myriad ligands and cells which regulate differentiation, and *in vivo* tissue injury models may induce lineage-independent endothelial marker expression in mesenchymal cells. By using an inducible Cre model to mark mesenchymal cells (*Scx-creERT/tdTomato* + ) prior to injury, we demonstrate that musculoskeletal injury induces expression of CD31, VeCadherin, or Tie2 in mesenchymal cells. VeCadherin and Tie2 were expressed in non-endothelial cells (CD31−) present in marrow from uninjured adult mice, thereby limiting the specificity of these markers in inducible models (*e.g.* VeCadherin- or Tie2-creERT). However, cell transplantation assays confirmed that endothelial cells (ΔVeCadherin/CD31+/CD45−) isolated from uninjured hindlimb muscle tissue undergo *in vivo* EndMT when transplanted directly into the wound without intervening cell culture using PDGFRα, Osterix (OSX), SOX9, and Aggrecan (ACAN) as mesenchymal markers. These *in vivo* findings support EndMT in the presence of myriad ligands and cell types, using cell transplantation assays which can be applied for other pathologies implicated in EndMT including tissue fibrosis and atherosclerosis. Additionally, endothelial cell recruitment and trafficking are potential therapeutic targets to prevent EndMT.

Endothelial-to-mesenchymal transition (EndMT) is a proposed process by which endothelial cells differentiate into mesenchymal cells^1^. This process appears to be initiated by tissue damage prompting the activation of pathways governed by transforming growth factor-β (TGF-β), in a mechanism similar to epithelial-to-mesenchymal transition^2^. Tissue healing disorders following injury including cardiac fibrosis^3^,^4^, atherosclerosis^5^, pathologic vein graft remodeling^1^,^6^, and heterotopic ossification^7^ have all been associated with endothelial-to-mesenchymal transition (EndMT).

A multitude of evidence has been collecting supporting the existence of EndMT. Despite the multitude of disorders in which EndMT has been implicated as a factor, unambiguous evidence of EndMT via lineage-tracing has remained elusive *in vivo* in the setting of tissue injury. This is due to the use of Cre drivers which lack specificity for endothelial cells^1^,^3^,^7^, non-inducible Cre systems which leave open the possibility of injury-induced promoter activity n^1^,^7^, and active immunostaining methods to identify endothelial cells which are unable to differentiate induced expression from lineage^1^,^3^,^5^,^7^. Additionally, because Tie2-cre or VeCadherin-cre label hematopoietic cells, it is not possible to differentiate circulating endothelial cells from circulating hematopoietic elements using these Cre drivers. This leaves open the possibility that circulating non-endothelial hematopoietic cells may migrate to site of wound injury and undergo mesenchymal differentiation.

*In vitro* experiments have also demonstrated that cells with hyperactive bone morphogenetic protein (BMP) signaling, as in fibrodysplasia ossificans progressiva, can undergo EndMT^7^,^8^,^9^. BMPs are part of the TGF superfamily, consistent with the proposed role of TGF-β signaling. However *in vitro* experiments, while supportive, are unable to represent the exact conditions of healing wounds.

In this study, we use a trauma-induced model of heterotopic ossification (tHO) to demonstrate that even in the absence of genetic BMP receptor hyperactivity, endothelial cells are capable of undergoing EndMT. We found that locally transplanted endothelial cells undergo EndMT in the wound site. Additionally, those endothelial cells which trafficked to the wound site after intravenous injection also underwent EndMT. These findings demonstrate that *a priori* endothelial cells are capable of undergoing EndMT, and that this process is not restricted to local endothelial cells. These findings have clinical import, as EndMT may be inhibited not only by targeting TGF-β signaling, but also by targeting endothelial cell recruitment.

## Results

### Cre-labeled mice suggest EndMT in a model of trauma-induced HO

**Figure 1:**
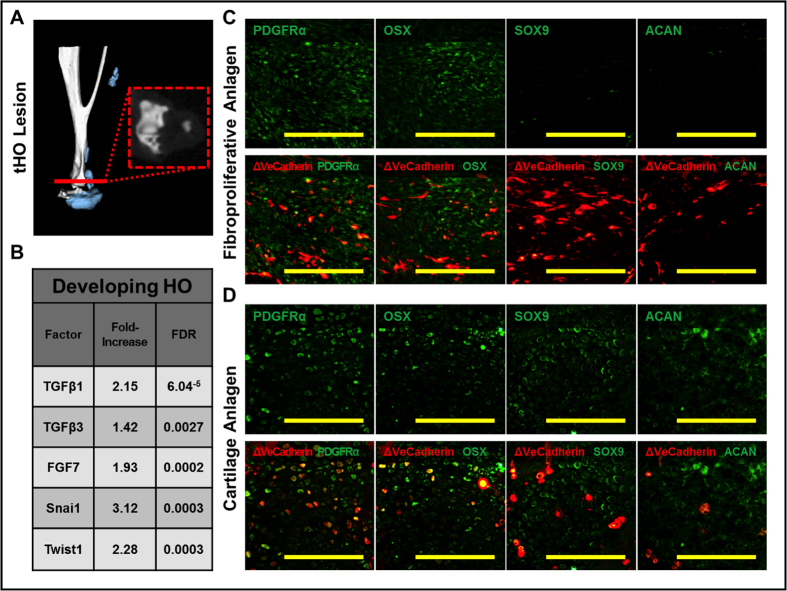
VeCadherin-cre-labeled mice suggest EndMT in a model of trauma-induced HO. (**A**) Burn/tenotomy results in trauma-induced HO (tHO) at the tendon transection site; (**B**) RNA Seq shows up-regulated transcript levels for Tgfβ, fibroblast growth factor (FGF), Snai1, and Twist1; (**C**) VeCadherin-cre/tdTomato lineage-tracing mice show presence of tdTomato + cells in the fibroproliferative region expressing PDGFRα, Osterix (OSX) but not SOX9 or Aggrecan (ACAN); D) VeCadherin-cre/tdTomato lineage-tracing mice show presence of tdTomato+ cells in the cartilage region expressing PDGFRα, Osterix (OSX), SOX9, and Aggrecan (ACAN).

Lineage-tracing using Tie2-cre has been previously performed suggesting that EndMT contributes to HO in the setting of local BMP4 injection^7^. Because the levels of BMP4 are supraphysiologic and do not represent wound conditions post-injury, we utilized a mouse model of trauma-induced HO (tHO) in which the Achilles’ tendon is transected and the mouse dorsum is burned^10^; tHO forms at the tendon transection site (Fig. 1A). This model closely represents the degree of injury incurred by patients with musculoskeletal trauma and burns who may develop tHO. RNA-Seq confirmed that the cartilage anlagen expresses several factors previously implicated in EndMT including Tgfβ, fibroblast growth factor (FGF), Snai1, and Twist1 (Fig. 1B). We next performed burn/tenotomy in mice labeled by VeCadherin-cre (VeCadherin-cre/tdTomato + ). In the absence of injury, tdTomato + cells mark vessel structures in these mice (Fig. 1C). We found that VeCadherin-cre did mark cells located within the fibroproliferative region and cartilage anlagen which precede maturation (Fig. 1C,D). Furthermore, VeCadherin-cre cells expressed the mesenchymal markers PDGFRα, Osterix (OSX), SOX9, and Aggrecan (ACAN) (Fig. 1C,D). PDGFRα^11^,^12^ has been used extensively as a mesenchymal marker, as has OSX^13^ during both chondrogenic and osteogenic differentiation. Furthermore, SOX9 and Aggrecan both are suggestive of chondrogenic differentiation. Importantly, these markers were not expressed by endothelial cells located in vessels at uninjured regions (Fig. S1). Taken together, these findings suggest that EndMT occurs during the progression of tHO.

### Trauma induces endothelial marker expression in non-endothelial cells

**Figure 2:**
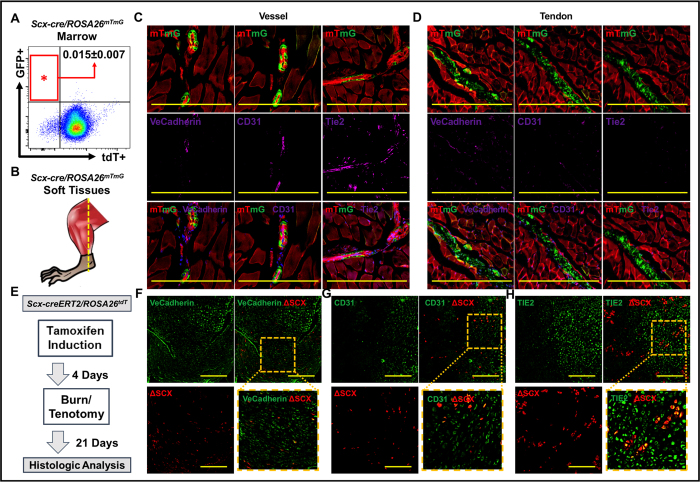
Trauma induces endothelial marker expression in non-endothelial cells. (**A**) Flow cytometry confirms relative absence of Scleraxis-cre cells in marrow (0.015% +/−0.007%); (**B**) Site of tissue harvest from Scx-cre/ROSA26^mTmG^ mice; (**C**) Immunostaining of vessel from Scx-cre/ROSA26^mTmG^ mice with VeCadherin, CD31, and Tie2 confirms that endothelial cells are not marked by the Scleraxis-lineage; (**D**) Immunostaining of tendon from Scx-cre/ROSA26^mTmG^ mice with VeCadherin, CD31, and Tie2 confirms that cells of the Scleraxis-lineage within the hindlimb soft tissue are not endothelial cells; (**E**) Experimental design to mark non-endothelial, mesenchymal cells using Scx-creERT2/tdTomato + mice; (**F**) tdTomato + cells (ΔSCX) from Scx-creERT2/tdTomato + mice express VeCadherin after injury; (**G**) tdTomato + cells (ΔSCX) from Scx-creERT2/tdTomato + mice express CD31 after injury; (**H**) tdTomato + cells (ΔSCX) from Scx-creERT2/tdTomato + mice express Tie2 after injury. Yellow scale bars = 200 um.

Although VeCadherin-cre mice provided evidence of EndMT, it was unclear whether tdTomato+/Sox9 or tdTomato+/Osx cells were initially mesenchymal cells which expressed VeCadherin, or endothelial cells which later expressed mesenchymal markers. Previous studies using BMP4 injection have shown similar results using Tie2-cre, with the same caveat of expression order. To demonstrate the possibility that trauma induces endothelial marker expression in non-endothelial cells we used Scleraxis as a marker of non-endothelial cells. Scleraxis is known to mark cells of the tendon and connective tissue lineage^14^,^15^,^16^,^17^. Flow cytometry confirmed that marked cells are present only to a small degree 0.015% +/−0.007%) in the marrow (Fig. 2A). Histologic analysis of uninjured muscle/tendon from Scx-cre/ROSA26^mTmG^ mice with co-staining for VeCadherin, CD31, and Tie2 confirms that cells marked by Scx-cre (GFP + ) are non-endothelial (Fig. 2B,C). Additionally, tendon marked by GFP expression in these mice does not stain with VeCadherin, CD31, or Tie2 further confirming that exclusion of endothelial markers from the Scx-cre lineage (Fig. 2D). To mark pre-injury non-endothelial cells expressing scleraxis, mice in which mesenchymal cells derived from the tenocyte lineage are marked by tdTomato + expression (*Scx-creERT/tdTomato*^*fl/fl*^) were induced with tamoxifen prior to injury and subsequently underwent burn/tenotomy (Fig. 2E). Vessels from uninjured sites expressed endothelial markers including VeCadherin, CD31, and Tie2, but were not marked by tdTomato expression in these mice (Fig. S2). Analysis of the cartilage anlagen three weeks after injury showed presence of tdTomato + cells. Even more striking however was that these cells expressed VeCadherin (Fig. 2F), CD31 (Fig. 2G), and Tie2 (Fig. 2H). Therefore, injury induces expression of endothelial markers in non-endothelial cells, thereby calling into question results using active endothelial marker expression immunostaining or non-inducible Cre systems to prove EndMT in the setting of trauma or other interventions which alter tissue homeostasis^1^,^7^.

### Locally transplanted endothelial cells undergo EndMT

*In vitro* experiments have demonstrated the ability of endothelial cells to undergo EndMT with addition of specific ligands or mutations^7^,^8^,^18^. However, the local wound environment differs from the *in vitro* setting in that multiple cells and multiple ligands are present which may alter the ability of any one ligand to initiate EndMT in contrast with *in vitro* experiments. Tamoxifen-inducible VeCadherin-creERT mice provide evidence supportive of EndMT, but may be confounded by the presence of labeled non-endothelial hematopoietic elements^6^. Flow cytometry experiments confirmed that the marrow contains Tie2+/CD45+/CD31− and VeCadherin+/CD45+/CD31− cells, suggesting that non-endothelial hematopoietic cells express Tie2 and VeCadherin even in adulthood (Fig. S3A,B). To avoid the influence of hematopoietic cells, transplantation of isolated veins from Tie2-cre labeled mice has been performed showing evidence of EndMT; however, the non-inducible Tie2-cre system leaves open the possibility of induced Tie2 expression in non-endothelial cells^1^.

**Figure 3:**
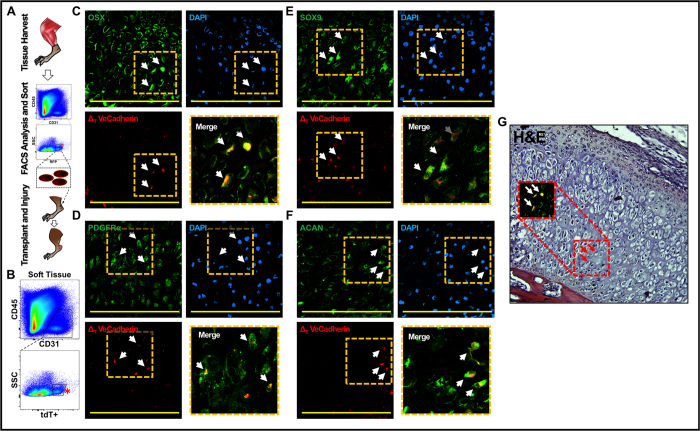
(**A**) Schematic showing cell transplantation experiments; (**B**) FACS sorting of endothelial cells (tdTomato+/CD31+/CD45−) from the hindlimb musculature of uninjured VeCadherin-cre/tdTomato + mice; (**C**) tdTomato + cells are present within the cartilage anlagen and express Osterix (OSX); (**D**) tdTomato + cells are present within the cartilage anlagen and express PDGFRα; (**E**) tdTomato + cells are present within the cartilage anlagen and express SOX9; (**F**) tdTomato + cells are present within the cartilage anlagen and express Aggrecan (ACAN); (**G**) H&E showing histologic character of tdTomato + cells undergoing EndMT with chondrocyte appearance and OSX expression as in (**C**).

To provide further support for EndMT *in vivo*, we used a series of cell transplantation experiments (Fig. 3A). Local endothelial cells (tdTomato+/CD31+/CD45−) from the hindlimb musculature of uninjured VeCadherin-cre labeled mice were isolated using FACS (Fig. 3B). The exclusion of bone or bone marrow during the tissue harvest process, and the selection of CD45−negative cells on FACS ensured that hematopoietic cells were excluded from the isolated fraction of endothelial cells. These *a priori* tissue-resident endothelial cells were then transplanted directly into the tendon transection site of unlabeled mice within 6 hours after burn/tenotomy with no intervening culture period. We were able to identify some tdTomato + cells present around areas suggestive of early vessel patterning (Fig. S4). Based on our findings with VeCadherin-cre labeled mice, we examined the cartilage anlagen of transplanted mice three weeks after injury and identified labeled (tdTomato+) cells within the anlagen. Immunostaining confirmed that these cells expressed OSX (Fig. 3C), PDGFRα (Fig. 3D), SOX9 (Fig. 3E), and ACAN (Fig. 3F), all mesenchymal markers. Additionally, H&E staining of these same histologic sections demonstrated regional and specific cellular morphologic changes corresponding to immunostained cells, providing evidence that the consequences of EndMT extend beyond changes in protein expression profile, and include changes in cell phenotype (Fig. 3G).

EndMT has also been implicated in atherosclerotic disease of the aorta^5^. Therefore, we cultured mature aortic endothelial cells (Cell Biologics) *in vitro* and injected these cells directly into the tenotomy site after burn/tenotomy (Fig. S5A). These cells, labeled with membrane-bound CellVue (CV+)^19^, were identifiable in the cartilage anlagen after three weeks, again demonstrating expression of Osterix (Fig. S5B) and Sox9 (Fig. S5C). Furthermore, these cells were in regions showing morphologic changes similar to those observed with the labeled cells isolated from muscle tissue. Taken together, these results suggest that the tissue environment following trauma supports EndMT in local endothelial cells.

### Circulating endothelial cells undergo EndMT after trafficking to the wound site

**Figure 4:**
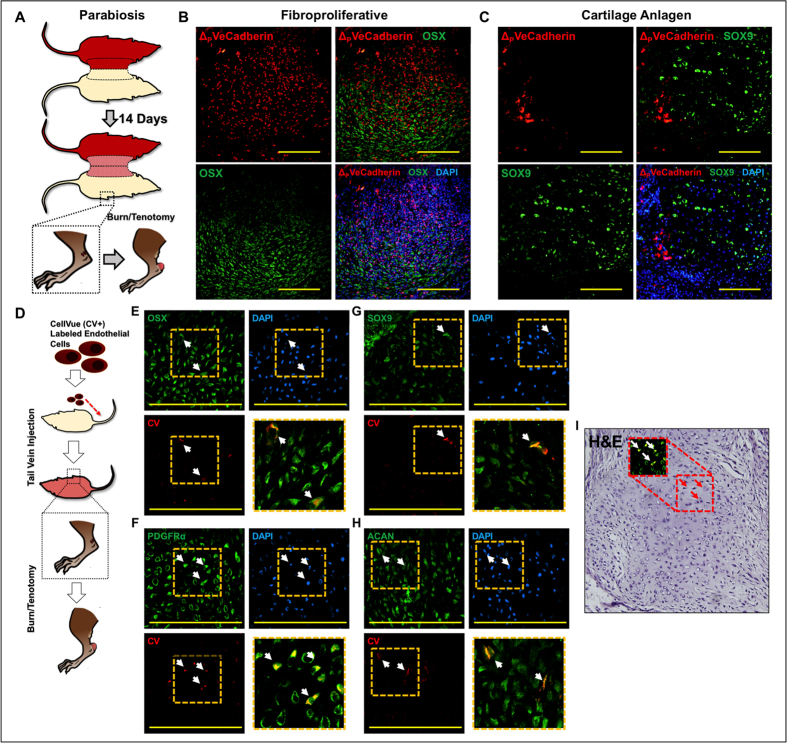
(**A**) Schematic showing parabiosis of VeCadherin-cre/tdTomato mouse with unlabeled wild type mouse and injury on unlabeled wild type mouse; (**B**) tdTomato + (ΔVeCadherin) cells in parabiosis model express Osterix (OSX); (**C**) tdTomato + (ΔVeCadherin) cells in parabiosis model express SOX9; (**D**) Schematic showing tail vein injection of cultured, Cell-Vue-labeled (CV+) endothelial cells after trauma; (**E**) CV + endothelial cells are present in the injured site and express Osterix (OSX) with merge image in lower right quadrant; (**F**) CV + endothelial cells are present within the cartilage anlagen and express PDGFRα with merge image in lower right quadrant; (**G**) CV + endothelial cells are present within the cartilage anlagen and express SOX9 with merge image in lower right quadrant; (**H**) CV + endothelial cells are present within the cartilage anlagen and express Aggrecan (ACAN) with merge image in lower right quadrant; (**I**) H&E showing histologic character of CV + cells undergoing EndMT with chondrocyte appearance and PDGFRα expression as in (**F**).

Next, we were interested in determining whether endothelial cells which traffic to the site of injury are capable of undergoing EndMT. This carries potential treatment conseqences, as blockade of endothelial cell recruitment may be a viable therapeutic strategy to prevent pathology-inducing EndMT^20^. Therefore, we sought to establish the possibility that circulating endothelial cells contribute to trauma-induced tHO using a parabiosis mouse model joining an unlabeled mouse with a VeCadherin-cre labeled mouse (Fig. 4A). Three weeks after injury to the unlabeled mouse, the tenotomy site was noted to have tdTomato + cells, which also expressed Osterix and Sox9 (Fig. 4B,C). This provided further evidence that circulating cells undergo EndMT at the wound site, but left open the possibility that circulating non-endothelial cells are induced to express endothelial markers in the wound site due to the altered tissue environment.

Therefore, we injected mature aortic endothelial cells into the mouse tail vein twelve hours after burn/tenotomy to determine whether *a priori* endothelial cells recruited early to the tenotomy site can undergo EndMT (Fig. 4D). In the fibroproliferative region and cartilage anlagen, injected endothelial cells were observed (CV+) expressing Osterix (Fig. 4E), PDGFRα (Fig. 4F), Sox9 (Fig. 4G), and ACAN (Fig. 4H). Again H&E staining confirmed a morphologic change in the appearance of marked cells (Fig. 4I). Taken together, these findings indicate that circulating mature endothelial cells are capable of undergoing EndMT and contributing to wound pathology, and that this process occurs early after injury.

## Discussion

In this study, we use *in vivo* cell transplantation assays in a model of musculoskeletal injury to verify the existence of EndMT. Transplantation of *a priori* endothelial cells directly into the injury site resulted in the expression of mesenchymal markers including PDGFRα^12^, Osterix (OSX)^13^, SOX9, and Aggrecan (ACAN). Furthermore, intravenous injection showed that endothelial cells which are recruited to the site of injury also undergo EndMT, suggesting that therapies which target cell recruitment may also be effective in limiting the pathologic contribution of endothelial cells. Interestingly, we found that injury can also induce endothelial marker expression in pre-defined mesenchymal cells, suggesting that studies examining EndMT using non-inducible Cre models must be cautiously interpreted.

EndMT has garnered increasing support in the literature^1^,^3^,^5^,^7^. Initially, studies utilized non-inducible Cre systems including VeCadherin-cre or Tie2-cre to show evidence of EndMT, as has been performed for fibrodysplasia ossificans progressiva (FOP), a genetic variant of heterotopic ossification^7^,^8^. However, these studies have been confounded by the possibility that cytokines and signaling mediators present within wounds can induce mesenchymal cells to express endothelial markers. By labeling mesenchymal cells *before* injury using the tamoxifen-inducible Scx-creERT/tdTomato mouse, we show that indeed tdTomato + mesenchymal cells express endothelial markers *after* injury. Previous studies have shown scleraxis to be a specific marker of mesenchymal cells in tendon and ligaments^16^. To further demonstrate that mesenchymal cells marked by Scx are not endothelial cells, we used a less restrictive non-inducible Cre model (Scx-cre) and showed that in the absence of injury, Scx does not mark endothelial cells. Furthermore, Scx-cre marked cells are present to only a small degree (<0.001%) in the marrow. Taken together, these findings indicate that non-inducible Cre models are confounded by endothelial marker expression in *a priori* mesenchymal cells, therefore supporting our use of cell transplantation techniques to verify EndMT.

Recently, Kolind *et al*. have studied the endothelial and mesenchymal contributions to BMP2-induced bone formation using Tie2-cre and αSMA-CreERT labeled mice^21^. They found that Tie2 primarily marked endothelial cells in this model; closer evaluation of their immunostaining shows some expression of SOX9 in Tie2-labeled cells. Although they did not find a significant reduction in bone formation when BMP2 was implanted in *Tie2-cre/Osx*^*fl/fl*^mice, they did show a trending reduction. Taken together with our findings, it remains possible that endothelial cells undergo EndMT, but that their contribution to pathology such as heterotopic ossification remains minimal. Furthermore, it is possible that the method of HO initiation – BMP2 implantation versus trauma in our model accounts for the observed differences. The model of musculoskeletal injury we use in this model results in trauma-induced heterotopic ossification (tHO) at the site of tendon transection. Previous studies have suggested that EndMT occurs in genetic variants of HO due to hyperactive bone morphogenetic protein (BMP) signaling; however, in addition to using Tie2-cre, a non-specific marker for endothelial cells, genetic variants of HO represent an exceedingly rare proportion of patients who develop this pathology. However, the occurrence of EndMT in tHO which occurs in the broader population of wounded patients without HO-inducing mutations suggests similar governing mechanisms for cellular differentiation. RNA-Seq analysis of tissue isolated from the model of tHO shows up-regulation of TGF-β, ACVR1, and Snai1, all factors previously implicated in EndMT^1^,^3^,^5^,^6^,^7^.

Direct *in vivo* endothelial cell transplantation resulted in the expression of mesenchymal markers including Osterix and Sox9, indicative of EndMT. EndMT has previously been demonstrated using isolated endothelial cells *in vitro* with the addition of specific cytokines to induce this differentiation process^22^. However, *in vitro* experiments cannot account for the myriad cell types and ligands which influence the tissue environment and EndMT. The series of cell transplantation performed in the tHO model demonstrate that trauma is sufficient to produce the signaling mediators required for EndMT to occur in an environment representative of trauma. The endothelial cells used for transplantation were isolated directly from the hindlimb musculature with gross anatomic exclusion of bone or marrow, and gating during FACS to exclude any possible hematopoietic elements present in vasculature on the basis of CD45 expression. Furthermore, routine histologic analysis showed that these same cells undergoing EndMT were not incorporated into vessels, but instead were located in regions showing signs of mesenchymal cell condensation. Therefore, findings of EndMT based on immunohistochemistry were corroborated with a phenotypic manifestation.

Finally, intravenous injection of endothelial cells also led to identifiable EndMT. This finding is significant for several reasons. First, it suggests that circulating endothelial cells are capable of homing to the injury site and contributing to pathology. Tamoxifen-inducible VeCadherin-creERT or Tie2-creERT models are unable to differentiate systemic from local components in the absence of parabiosis. Even with parabiosis, our analysis suggests that even in adulthood, the marrow contains non-endothelial hematopoietic cells (Tie2+/CD45+/CD31− or VeCadherin+/CD45+/CD31−), leaving open the possibility that a non-endothelial circulatory component is observed in these inducible Cre models. Although we did perform parabiosis and showed that systemically-derived VeCadherin-cre cells contribute to EndMT, we proceeded with cell transplantation because 1) non-inducible Cre systems may be confounded as mentioned earlier and 2) VeCadherin-cre may also label hematopoietic elements which respond to injury. By isolating aortic endothelial cells and injecting these intravenously 12 hours after injury, we show that endothelial cells present in the circulation can contribute to EndMT early after injury, opening the therapeutic possibility of targeting endothelial cell trafficking and recruitment.

Cell transplantation studies have obvious limitations, most notably that they cannot reliably determine the proportion of cells undergoing EndMT in the lesion. In addition, because a larger number of endothelial cells may be introduced than are normally present within the tissue, cell transplantation directly into the injury site may exaggerate the presence of EndMT. However, these studies are critically needed to verify the existence of EndMT because 1) non-inducible Cre models are confounded by expression of endothelial markers by mesenchymal cells after tissue injury, 2) VeCadherin and Tie2 label hematopoietic elements, and 3) *in vitro* studies while supportive are not representative of *in vivo* conditions with myriad ligands and cell types which variously influence EndMT. Furthermore, no studies have demonstrated the contribution of circulatory endothelial cells, which may serve as an additional target to prevent pathology associated with EndMT. We propose that EndMT is a pathologic process which contributes to tHO and that mechanisms which target early endothelial trafficking deserve further attention to mitigate EndMT. Future cell transplantation studies similar to this will be required to confirm these findings in alternative models of EndMT.

## Methods

### Animals

All animals were used according to the guidelines of the Institutional Animal Care and Use Committee (IACUC) of the University of Michigan, Ann Arbor, Michigan. All experimental protocols were approved by the University of Michigan IACUC (PRO# 5909). All burn/tenotomy injuries were performed in unlabeled wild-type C57BL/6 mice^10^. VeCadherin-cre/tdTomato + mice on C57BL/6 background served as muscle tissue donors for endothelial cell isolation. Scx-creERT/tdTomato + mice underwent burn/tenotomy after receiving post-natal tamoxifen injections to induce tdTomato expression in Scleraxis-expressing mesenchymal cells within the tendon.

### Burn/tenotomy injury model

Wild type C57BL/6 mice or mixed background Tg mice (aged 4–6 weeks) underwent hindlimb Achilles’ tendon transection with concomitant dorsal burn injury to induce heterotopic ossification^10^. Mice were anesthetized with isoflurane and depth of anesthesia was confirmed using toe-pinch prior to injury. Mice were administered buprenorphine for post-operative pain relief and monitored daily for the first 10 days after injury.

### Parabiosis model

Wild-type C57BL/6 and VeCadherin-cre/tdTomato + mice were surgically joined together at the lateral midline. Mice were monitored continuously for the first three days following joining, followed by twice daily evaluation for the next 11 days. Following a period of 14 days after joining, the unlabeled wild type mouse received burn/tenotomy injury.

### Flow cytometry analysis

Marrow was isolated from adult male mice for analysis of endothelial marker expression. Briefly, marrow was isolated from the femora and washed with PBS. Cells were then stained with anti-Tie2 or anti-VeCadherin antibody, anti-CD45 antibody, and anti-CD31 antibody. Samples were incubated for 30 minutes at 4 °C. Following incubation, samples were washed three times and filtered through a 45 micron mesh filter before being run on a FACSAria II (BD Biosciences) Cell Sorter at the University of Michigan Flow Cytometry Core. All analyses for endothelial cell markers were carried out in FlowJo.

### Endothelial cell isolation

Endothelial cells were isolated from the hindlimbs of VeCadherin-cre/tdTomato+ mice on C57BL/6 background (age 3–4 weeks). Specifically, hindlimb musculature was stripped from the bone and mechanically dissociated using sterile scissors. Care was taken to ensure that no bone, cartilage, or marrow components were included in the tissues. Tissues were digested for 120 minutes in 0.75% Collagenase 2 (Sigma-Aldrich) in Hanks Balanced Salt Solution (HBSS) at 37C under sustained agitation. Samples were filtered using successive 100− and 70-micron sterile strainers and digestion was quenched using equal parts 10% DMEM. Samples were centrifuges at 1000 rpm for 5 minutes before removing the supernatant and washing in HBSS.

Endothelial cells were then isolated from the digested muscle tissue using FACS. Briefly, the dissociated tissue was formed into a pellet using centrifugation (250 g for 10 minutes). The supernatant was discarded, and the tissue washed with PBS. Tissue was then stained with APC-conjugated anti-CD31 antibody and EF450-conjugated anti-CD45 antibody (eBioscience). Samples were incubated for 30 minutes at 4 °C. Following incubation samples were washed three times and filtered through a 45 micron mesh filter before being run on a FACSAria II (BD Biosciences) Cell Sorter at the University of Michigan Flow Cytometry Core. Endothelial cells marked by RFP+/CD31+/CD45− were collected. All analyses were carried out in FlowJo.

### Endothelial cell culture and CellVue Labeling

Commercially-prepared, mature C57BL/6-derived endothelial cells were purchased from Cell Biologics. Cells were labeled with CellVue per the manufacturer’s instructions. Cells were lifted from 10-cm culture plates with Trypsin-EDTA (0.5%) and centrifuged (250 g×10 minutes) to yield a pellet. After removal of the supernatant, the pellet was re-suspended in Diluent C with gentle pipetting to yield the 2 × Cell Suspension. Separately, a 2 × Dye solution was created by combining a volume of Diluent C equal to the 2x Cell Suspension volume and 4 uL of CellVue Dye. The 2 × Cell Suspension and the 2 × Dye Solution were then added together and immediately mixed with pipetting. Staining was allowed to proceed for 5 minutes and then stopped with addition of 10 mL of 10% FBS/DMEM. Cells were then centrifuged at 250 × g × 10 minutes. The pellet was washed with repeated centrifugation and suspension in PBS × 2. Cells were then re-suspended in Matrigel for cell transplantation or in PBS for intravenous injection.

### Cell transplantation and injection

Transplantation of cells into injured mice occurred within 12 hours after burn/tenotomy. For direct cell transplantation, cells were re-suspended in sterile Matrigel (50,000 cells/10 uL) which was injected directly into the tendon transection site of recipient mice. Injection was performed using a 27-gauge needle to minimize additional trauma to the transection site. For intravenous injection, cells were re-suspended in sterile PBS (150,000 cells/100 uL) which was injected into the tail vein of recipient mice.

### Immunofluorescence Microscopy

Tissue was processed for histologic evaluation using frozen sections. Tissue from the tendon transection site was collected 3 weeks after injury and fixed in 10% formalin for one hour at 4 C. Tissue was then incubated in 30% sucrose overnight at 4 C. Tissue was then embedded in optimal cutting temperature (OCT) media frozen at −80 C. Tissue was then ready to cut with thickness of 7 uM. Primary antibodies were anti-Sox9 (Santa Cruz) and anti-Osterix (Bioss). Secondary antibodies were anti-mouse AlexaFluor 488. Nuclei were stained with DAPI. Slides were mounted with Prolong Gold Antifade Reagent (Life Technologies) (Vector Labs) and images were obtained using a Nikon Olympus microscope.

## Additional Information

**How to cite this article**: Agarwal, S. *et al*. Local and Circulating Mature Endothelial Cells Undergo Endothelial to Mesenchymal Transition (EndMT) in Response to Musculoskeletal Injury. *Sci. Rep.* **6**, 32514; doi: 10.1038/srep32514 (2016).

## Supplementary Material

Supplementary Information
