# Supplementary material for: Local and Circulating Endothelial Cells Undergo Endothelial to Mesenchymal Transition (EndMT) in Response to Musculoskeletal Injury
Source: Sci Rep. 2016 Sep 12;6:32514. doi: 10.1038/srep32514 (PMC5018841; doi:10.1038/srep32514)
Supplement: Supplementary Information [file srep32514-s1.pdf]

Local and Circulating Endothelial Cells Undergo Endothelial to Mesenchymal Transition (EndMT) in Response to Musculoskeletal Injury

Shailesh Agarwal, MD[*,1]; Shawn Loder, BS[*,1]; David Cholok, BS[1]; Joshua Peterson, BS[1]; John Li, MD[1]; David Fireman, BS[1]; Christopher Breuler, BS[1]; Hsiao Sung Hsieh, DDS[1]; Kavitha Ranganathan, MD[1]; Charles Hwang, BS[1]; James Drake, BS[1]; Shuli Li, PhD[1]; Charles K. Chan, PhD[2]; Michael T. Longaker, MD[2]; Benjamin Levi, MD[1]

* These authors contributed equally to this manuscript

[1] Burn/Wound and Regenerative Medicine Laboratory, Department of Surgery, University of Michigan, Ann Arbor, MI 48109

[2] Department of Surgery, Stanford University, Stanford, CA 94305

SUPPLEMENTAL FIGURES

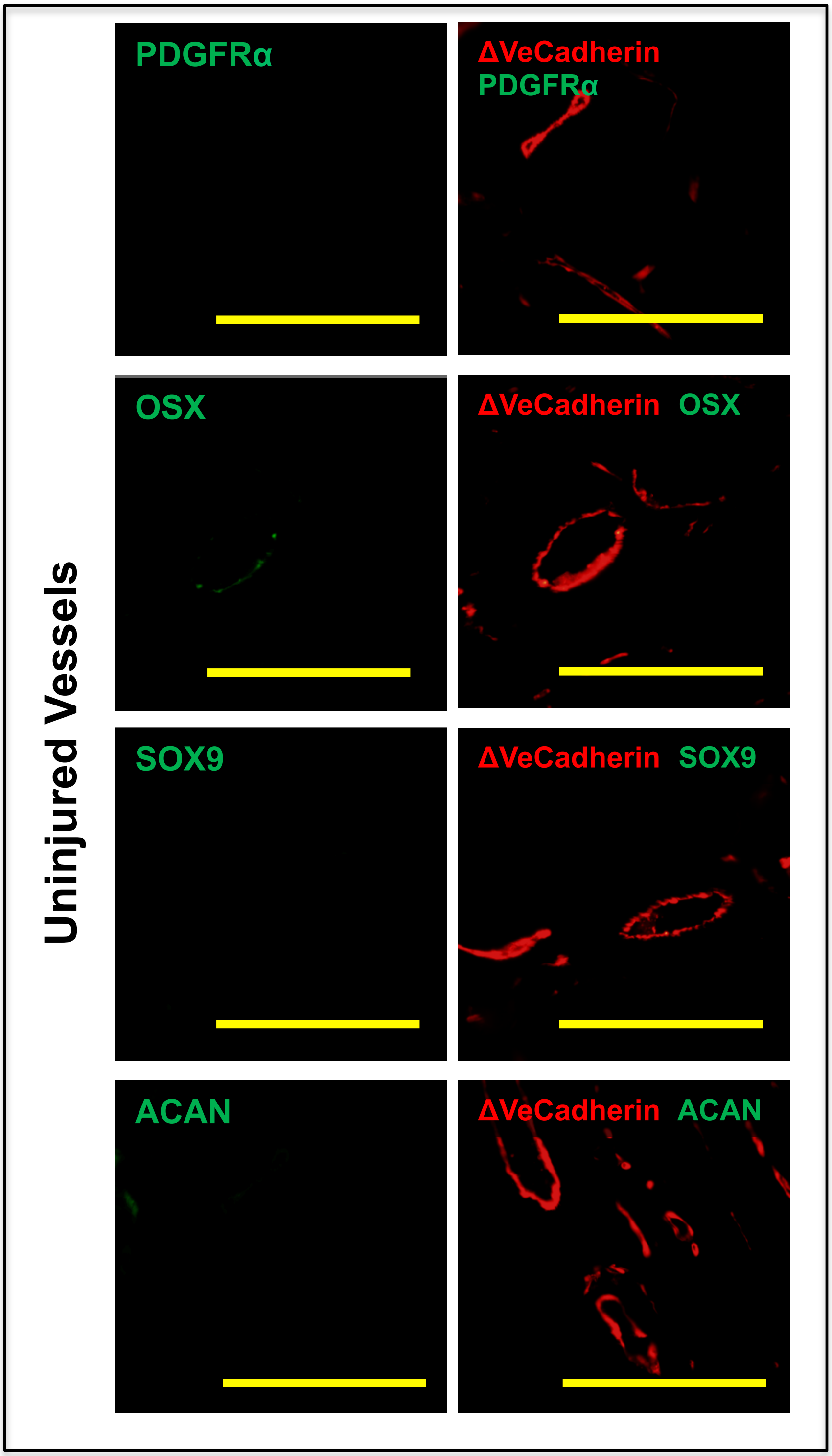

Fig S1. Endothelial cells (tdTomato; ΔVeCadherin) from uninjured vessels do not express mesenchymal markers including PDGFRα, Osterix (OSX), SOX9, or Aggrecan (ACAN). Yellow scale bars = 200 um.

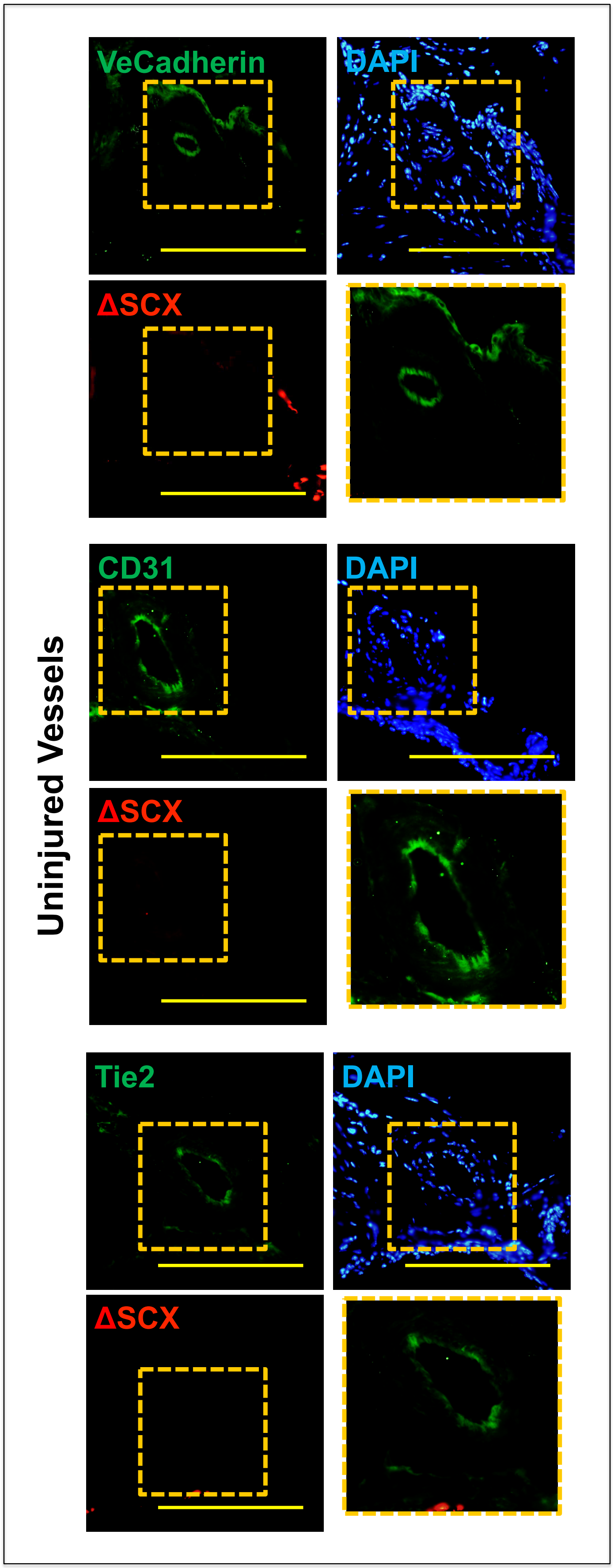

Fig S2. Uninjured vessels in the *Scx-creERT/tdTomato*$^{fl/fl}$ mouse express VeCadherin, CD31, and Tie2, but are not marked by tdTomato expression. Yellow scale bars = 200 um.

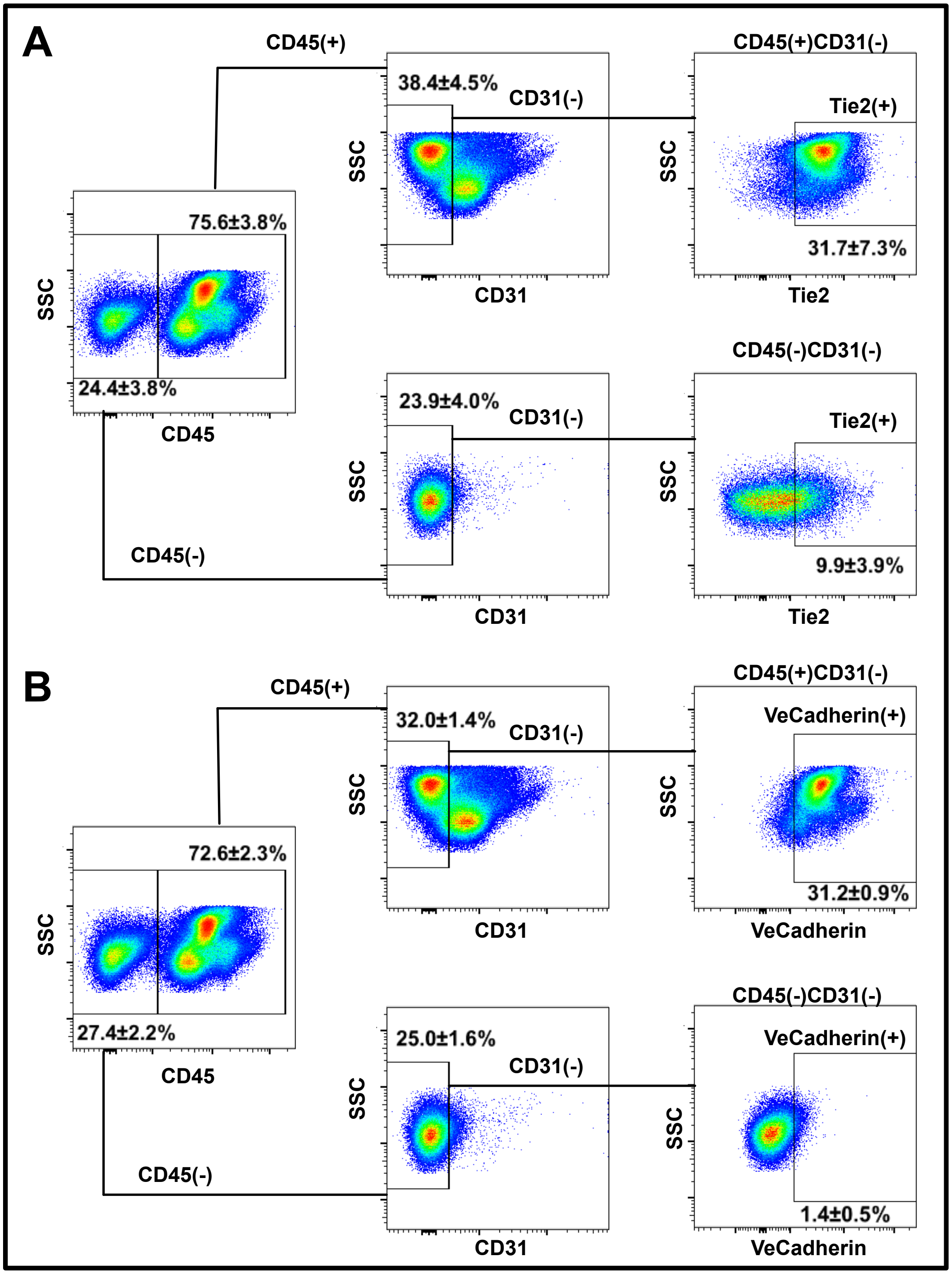

Fig S3. Non-endothelial cells in marrow express endothelial-cell markers. A) Non-endothelial cells in marrow express Tie2 (Tie2+/CD45+/CD31-: 31.7 +/- 7.3%; Tie2+/CD45-/CD31-: 9.9 +/- 3.9%); B) non-endothelial cells in marrow express VeCadherin (VeCadherin+/CD45+/CD31-: 31.2% +/- 0.9%; VeCadherin+/CD45-/CD31-: 1.4 +/- 0.5%) (n=3 mice/group)

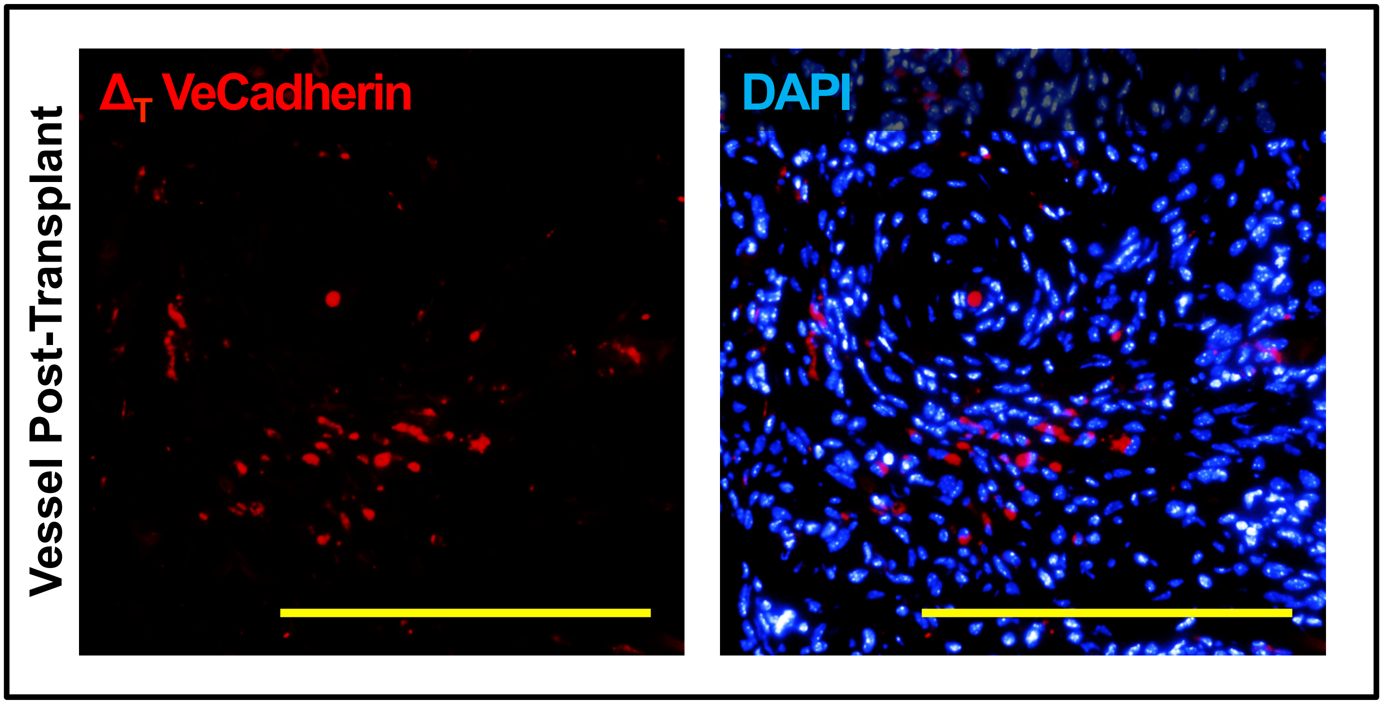

Fig S4. tdTomato+ cells isolated via FACS (tdT+/CD31+/CD45-) from the hindlimb musculature of uninjured *VeCadherin-cre/tdTomato*$^{fl/fl}$ mice are present around forming vessels post-transplant. All scale bars represent 200 um.

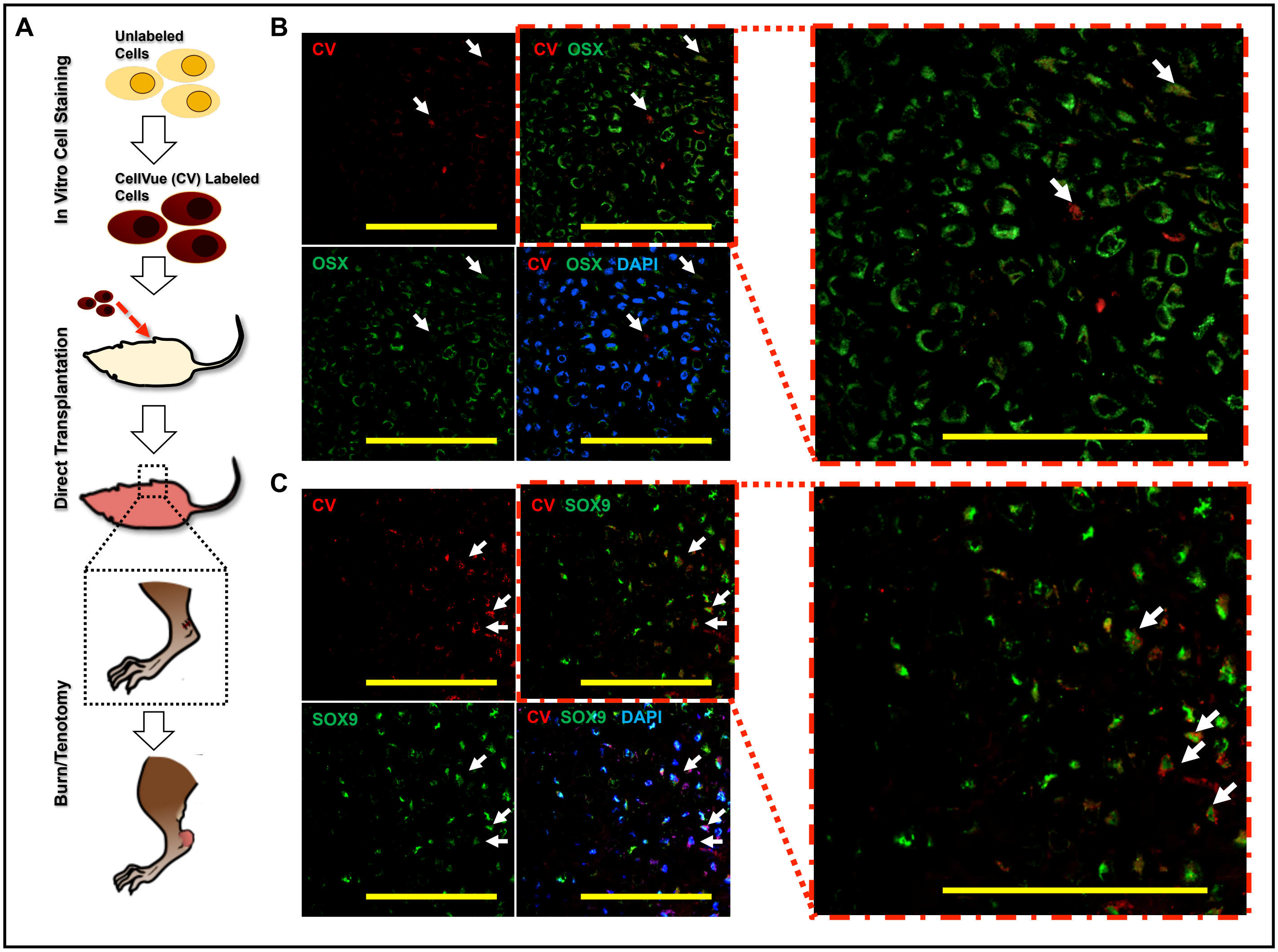

Fig S5. A) Schematic showing method of direct cell transplantation of cultured aortic endothelial cells with CellVue (CV) labeling; B) CV+ endothelial cells are present within the cartilage anlagen and express Osterix (OSX); C) CV+ endothelial cells are present within the cartilage anlagen and express SOX9. Yellow scale bars = 200 um.
